# Tailored SAPO-34/Graphite Adsorbent Composite Coatings on Aluminum Substrate for Energy Sustainable Sorption Technologies

**DOI:** 10.3390/polym17030260

**Published:** 2025-01-21

**Authors:** Davide Palamara, Edoardo Proverbio, Andrea Frazzica, Luigi Calabrese

**Affiliations:** 1 Department of Engineering, University of Messina, Contrada di Dio Sant’Agata, 98166 Messina, Italy; eproverbio@unime.it; 2 CNR-ITAE “Nicola Giordano”, Via Salita S. Lucia sopra Contesse 5, 98126 Messina, Italy; andrea.frazzica@itae.cnr.it

**Keywords:** zeolite, coating, S-PEEK, graphite, sorption, water vapor, adhesion

## Abstract

This paper explores a novel composite adsorbent coating applied to an aluminum support. This coating incorporates SAPO-34 and exfoliated graphite fillers within a sulfonate polyether ether ketone (S-PEEK) matrix, offering a promising avenue for energy-efficient adsorption technologies. Composite coatings, containing SAPO-34 zeolite as the primary adsorbent (80–95 wt.%) and exfoliated graphite as a conductive additive (5 wt.%) were produced. A drop-casting technique was employed to deposit the composite mixtures onto aluminum substrates. The coatings exhibited excellent adhesion to the metal substrate, as proved by a pull-off strength higher than 1.0 MPa. The morphological characterization revealed a uniform dispersion of both additives within the host material. To evaluate their adsorption/desorption behavior, equilibrium water vapor adsorption isobars were determined at a constant pressure of 11 mbar across a 30–120 °C temperature range. The adsorption/desorption tests showed the composite coatings reached 26–30% water uptake, indicating that the matrix did not obstruct the water vapor mass transfer, and the zeolite exhibited active participation in the adsorption/desorption process. These results suggest that this material may be a promising candidate for energy saving systems.

## 1. Introduction

The growing global demand for sustainable and efficient energy solutions has driven significant research interest in sorption technologies [1,2,3]. These technologies, which harness the adsorption and desorption properties of materials to store and release energy, offer promising alternatives to traditional refrigeration and heating systems [4,5].

Sorption technologies offer a promising avenue for sustainable and energy-efficient solutions, particularly in thermal energy storage and cooling/heating applications. These technologies leverage the ability of certain materials, known as solid sorbents, to adsorb and release specific substances (i.e., working fluid), such as water vapor, alcohols, and other refrigerants [5]. In close working cycles, when heat is applied to the system, the solid sorbent releases the working fluid, which needs to be condensed and stored in a separate vessel. The released heat of condensation can be either released to the surrounding environment or used to provide heating to the user. Conversely, the working fluid’s evaporation and adsorption by the solid sorbent can provide cooling or heating to the user through the evaporator or adsorber, respectively, due to the exothermic adsorption reaction. This process can be continuous for an adsorption heat pump/chiller or discontinuous for long-term adsorption thermal storage [6,7].

Among the various solid sorbents explored, SAPO-34, a crystalline microporous aluminophosphate, has emerged as a leading candidate for operation under closed-cycle conditions, due to its relevant adsorption capacity, thermal stability, and selectivity [8,9].

Solid sorbent cycled during operation from a desorption temperature (i.e., range 70–90 °C) to an adsorption temperature (i.e., range 20–40 °C), integrated inside efficient heat exchangers, maximize the heat and mass transfer efficiency process. The most basic and cost-effective approach involves the use of unconsolidated bed adsorbent granules, placed directly within exchanger fins without a binding agent [10]. This method exhibits good mass transfer but suffers from poor heat transfer due to the limited grain–exchanger contact, low adsorbent conductivity, and the resulting low thermal conductivity within the bed [11].

Differently, consolidated bed configurations, achieved through direct synthesis [12,13] or binder use (usually polymeric) [14,15], offer improved heat transfer at the coating/metal interface, leading to reduced cycle times [16,17]. However, this advantage is offset by the potential limitations in mass and heat transfer caused by a polymeric binder and low bed porosity [18], which can impact the adsorption capacity and efficiency.

Limited heat and mass transfer within the adsorbent restricts the sorption technology performance by extending the cycle times and reducing the achievable heating/cooling power [8]. Maximizing the adsorbate diffusion and heat transfer is therefore critical in binder-based adsorbent composite coatings.

With this concern, in recent years, there has been a growing focus on tailoring and developing composite sorbents that combine the advantages of zeolite with the unique properties of other materials that act as functional binders [18,19].

Binder selection for adsorbent composite coatings necessitates careful consideration of properties like the thermal conductivity and adsorbate permeability. Specific studies have explored different binder systems to address these challenges. For instance, polyvinyl alcohol has been used as a binder to prepare dense mechanically stable coatings of AlPO-18 on an aluminum substrate [20]. However, for thicker layers, improvements in both the mass and heat transfer are necessary. Further studies addressed silane-based composite coatings, evidencing suitable sorption capacity for adsorption heat pump applications [21,22]. Similarly, studies using a 2-hydroxyethyl ether binder and SAPO-34 filler have identified mass transfer as a key hindering factor in the adsorption/desorption process, regardless of the coating thickness [23]. Therefore, water vapor-permeable matrices, such as SAPO-34/S-PEEK or PDMS-based composites, offer promising solutions due to their desirable mechanical and physicochemical properties [24,25,26,27].

Due to the low thermal conductivity of an S-PEEK polymer used as a binder, one such promising approach could involve the integration of SAPO-34 with graphite, a highly conductive and thermally stable material. By creating composite coatings of solid sorbent and graphite, researchers aim to enhance the overall performance of sorption systems, including improved adsorption kinetics, increased thermal conductivity, and enhanced mechanical stability [28,29].

In this work, a novel adsorbent coating is developed, consisting of SAPO-34 and exfoliated graphite fillers dispersed in a sulfonated polyether ether ketone (S-PEEK) matrix. This innovative approach aims to investigate a new class of sorbent coatings characterized by effective water vapor capacity, a key factor for energy efficiency, coupled with enhanced thermal conductivity, facilitating rapid heat transfer and accelerating the sorption/desorption cycle. In particular, composite materials differing in terms of the zeolite adsorbent filler content (SAPO-34: 80–95 wt.%) and the conductive filler content (exfoliated graphite: 0–10 wt.%) were prepared and coated onto aluminum substrates using a drop-casting technique. The mechanical properties of these coatings were assessed through pull-off and scratch tests, demonstrating suitable adhesion to the metal substrate. To evaluate the adsorption and desorption capabilities of the composite material, water vapor adsorption isobars were measured at a constant pressure of 11 mbar over a temperature range of 30–120 °C. These findings demonstrate that the polymer binder does not hamper the water vapor molecules’ transport across the coating, and the zeolite filler actively participates in the adsorption and desorption processes.

## 2. Materials and Methods

### 2.1. Materials

The PEEK polymer, with a glass transition temperature between 143 °C and 160 °C and a melting range of 338 °C to 348 °C, from HEROFLON S.p.A (Collebeato, Italy), in 0.5–1 mm granules was used as key constituent material for the sulfonated thermoplastic matrix. Concentrated sulfuric acid (H_2_SO_4_, 98% purity, obtained from Sigma-Aldrich, St. Louis, MO, USA) was chosen for dissolving PEEK. N,N-Dimethylformamide (N,N-DMF), supplied by Honeywell, was chosen as the solvent for both titration and coating preparation, with a minimum purity of 99.8%. To enhance the performances of the composite coatings, exfoliated graphite (EG) supplied by TIMREX C-THERM 002 TIMCAL Ltd. (Bodio, Switzerland) was utilized as a filler material. This EG is characterized by a bulk density of 0.04 g/cm^3^ and a specific surface area of 25 m^2^/g. Finally, SAPO-34 zeolite (AQSOA Z02) from Mitsubishi Plastics Inc. (Tokyo, Japan), with a primary grain size of 5–10 µm and a pore size of 0.38 nm, served as the absorbent filler.

### 2.2. Sulfonated PEEK Preparation

PEEK’s hydroquinone unit, activated for electrophilic substitution by ether linkages, preferentially accepts sulfonic groups (SO3H-) between its two ether bonds during low-temperature sulfuric acid treatment [30]. This work followed the sulfonation method described in [31]. In particular, 3 g of pre-dried (100 °C, 12 h) PEEK were dissolved in a round-bottom flask containing 25 mL of concentrated sulfuric acid (98%) under continuous stirring at room temperature (25 °C) up to 48 h. The mixture was then slowly dripped into ice-cold deionized water to quench the reaction and precipitate the sulfonated polymer. The precipitate was recovered by filtration then washed thoroughly by using distilled water until the filtrate tested neutral on pH-indicator paper, and dried in a lab oven at 60 °C for 24 h.

### 2.3. Composite Coating Preparation

According to [31], the composite coatings were created by drop-casting S-PEEK polymers and fillers dissolved in DMF onto Aluminum 6061. In particular, S-PEEK polymers (~0.2 g) were dissolved in about 2 g of DMF. Zeolite and graphite fillers at different ratios were gradually added to the solution, stirred at room temperature for 15 min, and drop-cast on aluminum substrate. The layer was dried at 60 °C for 12 h, resulting in coatings less than 0.50 mm thick.

The various composite batches under study were distinctly identified through a coding system. Each of these batches was designated with the common root SP-, followed by two dedicated numbers denoting the zeolite and graphite filler content in weight percentage (wt.%). To signify the graphite content, the prefixes G were utilized, whereas Z denoted the zeolite filler content. For instance, the code SP-Z90-G5 was employed to represent an S-PEEK composite coating composed of 90 wt.% zeolite filler and 5 wt.% graphite filler. This standardized coding approach streamlined the identification and differentiation formulation for the composite batches, enabling clear categorization based on their specific composition. The details are shown in Table 1.

### 2.4. Coating Characterization

For the analysis of the chemical features of the composite coatings, a PerkinElmer Spectrum Two Fourier-transform infrared (FTIR) spectrometer was utilized. This instrument operated in ATR mode, scanning a broad spectrum ranging from 450 to 4000 cm^−1^, with a resolution of 4 cm^−1^. The resulting data were represented in the form of spectra exhibiting absorbance (%) against wavenumber (cm^−1^), in the wavenumber range 1800–450 cm^−1^ providing a comprehensive insight into the molecular composition and characteristics of the S-PEEK composite coatings under examination.

With a focus on assessing the thermal stability, thermogravimetric analysis (TGA Q600, TA Instruments) was performed. First, 3–5 mg of composite coating, in a platinum crucible, were analyzed under air flow (100 mL/min). After a 20 min preheating at 120 °C, in order to remove moisture, the samples were cooled to 100 °C. Then, a temperature ramp from 100 °C to 800 °C (5 °C/min) was applied.

To assess the mechanical behavior of the coating under mechanical stress, two tests were conducted: a Clemen scratch test and a pull-off test.

Concerning the former, the scratch resistance for all composite sorbents was evaluated by a scratch tester (Elcometer 3000 manual Clemen). This instrument complies with the EN ISO 1518-1 standard [32] for scratch testing. The engraver consists of a hemispherical ball-shaped tip made of AISI 304 stainless steel with a 500 µm diameter. This tip is mounted on a truncated conical support, also made of AISI 304 stainless steel, with a 45-degree angle and a height of 1000 µm. The Clemen tester creates a scratch on the coating surface by applying a controlled force that increases with each test run. The sample is then moved at a constant speed of 10 mm/s. The scratching process stops automatically after the sample has moved a set distance of 40 mm. To determine the critical scratch load of the coating, the test was repeated on each sample with a varying load (in the range 750–2000 g). Following the scratch test, in order to assess the point at which the applied force caused significant damage to the coating, the resulting grooves were examined under a 3D digital optical microscope (KH8700 by Hirox, Tokyo, Japan) to measure their width and depth.

The investigation into the adhesive strength of the composite coating on the aluminum substrate was performed by pull-off test. A PosiTest AT-M pull-off tester (DeFelsko, Ogdensburg, NY, USA) was used as the testing set-up. It was employed to determine the force required for detaching a 10 mm diameter aluminum dolly that was previously glued onto the adsorbent composite coating. Maintaining the integrity of the test, a custom-designed clamping fixture was utilized to secure the sample in place, ensuring a direct and uniform pull during the examination process.

To assess the water vapor sorption capacity of the coatings, a thermogravimetric dynamic vapor system (Surface Measurements Systems DVS Vacuum) was used. This instrument is equipped with an electromagnetic balance (sensitive to 0.1 micrograms) able to measure weight changes while controlling the amount of water vapor surrounding the sample during time. The experiment involved exposing all the different composite coatings (which contained varying amounts of graphite filler and zeolite filler, from 80% to 95% by weight) in isothermal conditions: constant temperature (30 °C) at varying water vapor partial pressures (P/P_0_ = 0–90%). Finally, the equilibrium water uptake (W) was determined at each pressure step according to the following formula:

(1) $$W\left(wt.\;\%\right)=\frac{m\left(p_{H_{2}O,}T_{s}\right)-m_{0}}{m_{0}}\;\cdot 100,$$

where $m\left(p_{H_{2}O,}T_{s}\right)$ (g), refers to the weight of the sample when it reaches equilibrium with water vapor at a specific pressure p_H2O_ and temperature *T_s_*. In contrast, *m*_0_ (g) is the weight of the sample after all the water has been removed (dry weight).

## 3. Results and Discussion

**Figure 1:**
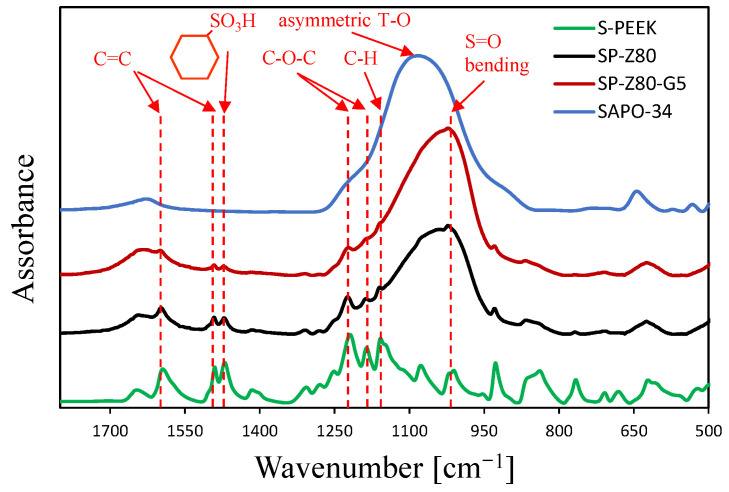
Comparison of the FTIR spectra of the S-PEEK polymer and the two zeolite coatings with and without graphite (SP-Z80 and SP-Z80-G5) in the wavenumber range 1800–450 cm^−1^.

Figure 1 shows the FTIR spectra of the zeolite composite coating with and without the graphite filler. As the reference spectrum, neat S-PEEK polymer was also added. The S-PEEK spectrum shows a characteristic peak at 1647 cm^−1^, indicating the aromatic stretching vibration of the CO bond [33]. Furthermore, the three peaks at 1595, 1487, and 1411 cm^−1^ correspond to the vibration of the aromatic ring [34]. Additionally, the peak at 1305 cm^−1^ is attributed to the C-C(=O)-C bond. The two peaks observed at 1276 and 1186 cm^−1^ can be ascribed to the C-O-C asymmetric stretching of the aryl ether group [35]. The peak at 1151 cm^−1^ is associated with the aromatic hydrogen bonds, while the peaks at 924 and 832 cm^−1^ are related to the diphenyl ketone and aromatic hydrogen bands, respectively [36]. Furthermore, The S-PEEK spectrum exhibits additional absorption peaks compared to the PEEK spectrum. These new peaks include an asymmetric O=S=O stretching peak at 1248 cm^−1^ and peaks at 1076, 1022, and 705 cm^−1^ attributed to the symmetric O=S=O stretching [37], S=O bending, and S-O stretching of the sulfone group [38], respectively.

Sulfonation causes the aromatic C-C vibration peak to split into two peaks: the former at 1490 cm⁻^1^ and a second one (that emerges due to sulfonation) at 1469 cm⁻^1^ [39]. This splitting of the aromatic C-C band is indicative of the introduction of the sulfonic group onto the benzene ring [40]. Therefore, spectral analysis indicates successful sulfonic group attachment to the PEEK backbone, confirming the suitability of the chosen synthesis time [41].

Observing the spectrum of the filled S-PEEK coatings (SP-Z80 and SP-Z80-G5), some of these characteristic peaks are still visible below the prominent SAPO-34 peaks. According to [42], within this specific wavenumber range, SAPO-34 exhibits a prominent broad peak centered around 1100 cm⁻^1^. This peak is associated with the asymmetric stretching vibrations within the tetrahedral T-O units (where T can represent silicon (Si), phosphorus (P), or aluminum (Al)). Given the high filler content (80%) and low matrix content (20%) of the analyzed coating, these peaks significantly influence the overall spectral profile of the composite coatings. Furthermore, the incorporation of exfoliated graphite filler results in a more pronounced peak at approximately 1640 cm⁻^1^. This peak surpasses the intensity of the adjacent peak at 1595 cm⁻^1^, which is attributed to the S-PEEK matrix. This enhancement is likely due to the presence of a peak characteristic of exfoliated graphite, specifically, the in-plane vibration of aromatic C=C stretching at around 1630 cm⁻^1^, as reported in reference [43]. Furthermore, the increased peak intensity for the SP-Z80 sample related to the S-PEEK matrix (e.g., at 1248 cm^−1^) likely originated from a greater abundance of the polymer matrix within its structure, compared to the SP-Z80-G5 one. This higher polymer content results in a stronger overall signal in the FTIR spectrum at this specific wavenumber.

**Figure 2:**
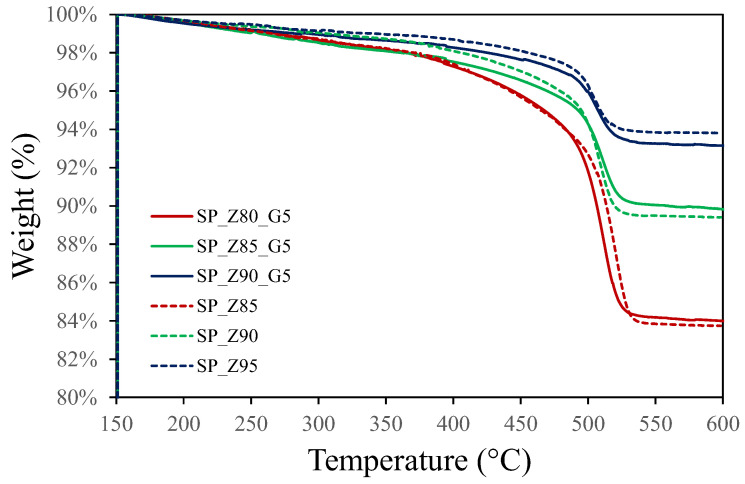
Thermo-gravimetric plot of weight vs. temperature of S-PEEK coatings with various content of zeolite and exfoliated graphite in the range 150–600 °C.

In Figure 2, the thermogravimetric analysis (TGA) curve of the composite S-PEEK coatings reveals the typical three-step degradation pattern of sulfonated matrix. A first step, is related to the initial weight loss, occurring at temperatures below 150 °C. This step, not identifiable in Figure 2, is likely attributed to the evaporation of residual solvents or moisture [35]. The second one, related to a shoulder in the mass loss trend, observed at intermediate temperatures (300–350 °C), suggests the onset of thermal degradation. This can be ascribed to the de-sulfonation reaction of –SO_3_H groups, which results in the release of SO_2_ and H_2_O [44]. In the final stage of degradation, the polymer chain undergoes substantial weight loss above 500 °C, indicating the complete breakdown of the polymer backbone. Oxidative pyrolysis, in which the polymer’s backbone structure breaks down due to exposure to oxygen at high temperatures, is the cause of this sudden drop [45]. Based on these findings, it can be concluded that the composite zeolite/graphite adsorbent coatings exhibit remarkable thermal stability up to approximately 300 °C. This temperature range exceeds the typical operating temperatures required for activating adsorbent fillers or powering adsorption heat pump cycles, making this class of material a promising candidate for applications in these fields.

The adhesion strength of composite coatings with varying filler contents was evaluated by pull-off tests.

**Figure 3:**
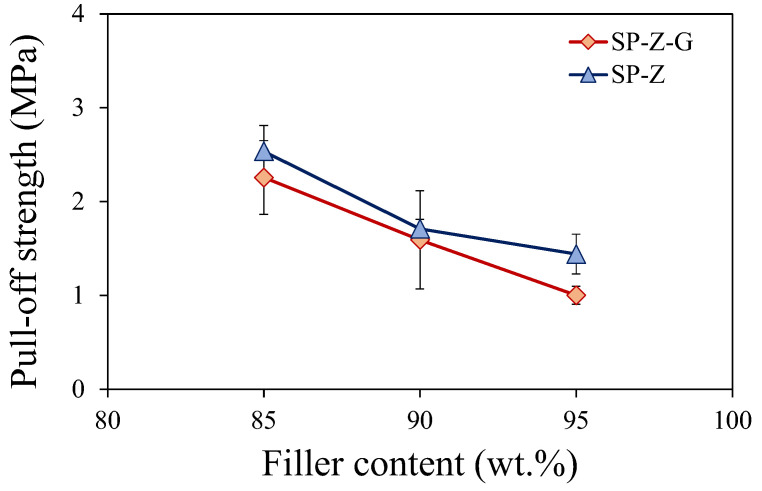
Pull-off adhesion strength of composite coatings at varying filler contents.

To gain a deeper understanding of the combined effect of graphite and zeolite fillers on the adhesive properties of the coating, a graph was created to illustrate the relationship between the pull-off strength and the total filler content (Figure 3). The total filler content is defined as the sum of the zeolite and graphite filler percentages within the coating formulation. For example, coatings with an 85% filler content encompassed batches SP-Z85 and SP-Z80-G5. Both these batches exhibited a 15% matrix content, representing the binder phase that provides cohesive strength and adhesion within the composite coating.

All composites exhibited strong adhesion, indicating a suitable interaction among the aluminum support, fillers, and polymer binder. However, the adhesion strength decreased slightly as the filler content increased. Even with lower adhesive properties, composites containing 95 wt.% filler (SP-Z95 batch) demonstrated significantly higher pull-off strength values (1.57–1.75 MPa) compared to the literature reports [46].

Employing an exfoliated graphite filler instead of a zeolite filler led to a reduction in the adhesive strength, especially when the filler content was increased. This phenomenon can be attributed to the difference in the surface properties and interfacial interactions between the two types of fillers and the polymer matrix.

Zeolite fillers, with their porous reactive structure and high surface area, tend to promote strong interfacial bonding with the polymer matrix through mechanisms such as mechanical interlocking and hydrogen bonding [47,48]. This leads to enhanced adhesion and improved mechanical properties of the composite material.

On the other hand, exfoliated graphite fillers, despite their high aspect ratio and potential for improving mechanical properties, may exhibit weaker interfacial adhesion with the polymer matrix. This could be due to the relatively smooth surface of exfoliated graphite, compared to zeolite, which slightly limits the opportunities for mechanical interlocking, and the lack of strong polar functional groups that can facilitate hydrogen bonding or other types of chemical interactions.

As the filler content increases, the influence of the filler’s surface properties and interfacial interactions becomes more pronounced. The lowest pull-off strength was observed at higher zeolite filler content levels. For example, the pull-off strength decreased from 1.44 MPa for SP-Z95 to 1.00 MPa for SP-Z90-G5. This indicates that a reduction of 30% was evidenced, although, according to [49], this value suggests an adhesion strength still higher than a threshold equal to 0.80 MPa. This finding suggests that, despite exhibiting the lowest pull-off strength, the adhesion strength of the SP-Z90-G5 batch remains sufficient for effective adsorption technologies, highlighting the potential for optimizing the coating parameters to enhance adhesion while maintaining desirable adsorption properties.

**Figure 4:**
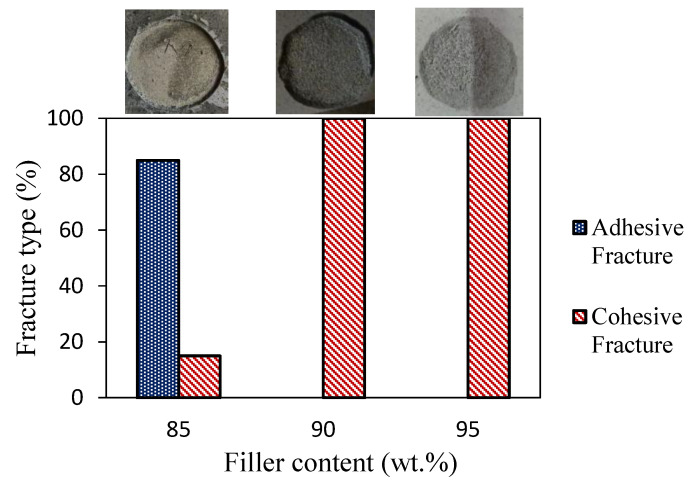
Cohesive and adhesive fracture type at varying filler contents for graphite-filled zeolite adsorbent coatings.

Further information can be gained by evaluating the failure mechanisms’ evolution at varying filler contents. Figure 4 compares the adhesive and cohesive fracture type occurring during the pull-off test at varying filler contents for the graphite-filled zeolite coating.

At a low filler content (85 wt.% filler), a mixed adhesive/cohesive failure mechanism was observed, with the adhesive failure dominating over the cohesive one (85% and 15%, respectively). Fracture initiation occurred at the coating/metal substrate interface, primarily progressing along the interface but occasionally extending into the coating bulk, resulting in partial cohesive fracture. However, with the increasing filler content, the cohesive failure became dominant. This shift can be attributed to the reduced S-PEEK matrix content in the 90% and 95% filled samples, which weakened the interfacial adhesion between the matrix and the zeolite/graphite fillers [21]. Consequently, the cohesive force within the coating is reduced, making it more susceptible to crack propagation through the bulk, despite the aluminum coating interface [46].

**Figure 5:**
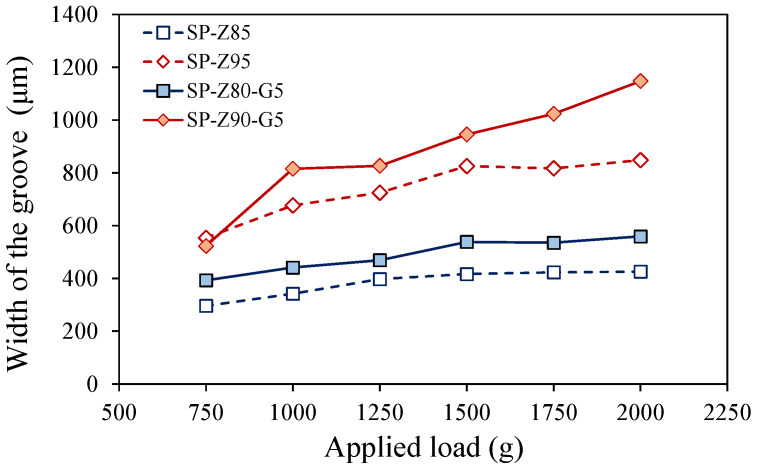
Measured groove width due to scratch test on composite coatings with varying filler contents subjected to different applied loads.

Furthermore, in order to better evaluate the adhesive and cohesive properties of the coating, the scratch resistance of the coatings was evaluated. The scratch widths of composite coatings containing 85 wt.% and 95 wt.% filler were evaluated under different applied loads, as depicted in Figure 5. To assess the influence of the exfoliated graphite, coatings with identical filler content (e.g., 85 wt.%) were produced with and without exfoliated graphite inclusions. A wider scratch width generally signifies reduced scratch resistance within the coating.

The SP-Z85 batch, containing no graphite and less filler, exhibited the best scratch resistance. Substituting 5 wt.% zeolite with 5 wt.% graphite (SP-Z85 to SP-Z80-G5) resulted in wider grooves. This trend persisted for coatings with 95 wt.% filler. Graphite’s lower density compared to zeolite means a larger amount of exfoliated graphite is added for the same weight, reducing the adhesion. Despite exfoliation, zeolite’s porous structure with more hydroxyl groups provides better interaction with S-PEEK’s polar groups, leading to stronger interfacial adhesion and higher cohesive force compared to the graphite-containing coatings.

Furthermore, the groove width is significantly influenced by the coating constituents. For all the coatings, the groove width increased with an increase in the applied load, reflecting a progressive reduction in scratch resistance under higher mechanical stress. This trend, identifiable by the slope of the scatter data trend, is particularly pronounced for batches at higher filler contents (SP-Z90-G5 and SP-Z95), indicating a more rapid increase in the scratch sensitiveness under a high applied load. For example, these batches exhibit a linear slope approximately 2.2 to 3.2 times higher than that of their corresponding Z80 counterparts.

In particular, the SP-Z90-G5 batch coating characterized by 95 wt.% of filler (90 wt.% SAPO-34 and 5 wt.% EG) consistently exhibits the widest grooves across all applied loads, suggesting a relatively weaker cohesive strength. This can be explained by considering that the matrix has a key role in holding together the solid particles of zeolite and graphite. The matrix acts as a glue, connecting these particles and ensuring the structural integrity of the coating. When the composite coating contains a high proportion of filler materials, the matrix, which acts as a binding agent, has a low content.

This amplifies a reduction in the cohesive properties of the coating, resulting in a decrease in its ability to resist to internal fracture. This reduction is attributed to the limited interfacial adhesion between the individual constituents (filler particles and matrix) within the bulk of the composite material. Consequently, the SP-Z90-G5 coating exhibits increased sensitivity to scratch-induced damage, leading to the formation of a wider and deeper groove compared to other coatings. This discrepancy in scratch resistance for high filler content coatings becomes more pronounced as the applied load increases. At lower applied loads (e.g., 750 g), the scratch resistance capacities of batches SP-Z90-G5 and SP-Z95 can be considered comparable. This aspect could be justified considering that for high filler content coatings, at low loads, the material might deform elastically or with minor plastic deformation (the coating is characterized by a lower amount of polymer matrix). These initial deformations might be relatively insensitive to small material variations. Instead, at higher loads, the deformation becomes more severe (e.g., plastic flow, crack initiation). In this regime, material differences can lead to significantly different deformation behaviors and, consequently, varying levels of scratch resistance. In fact, the dominant deformation mechanisms change with the increasing load. At low loads, the material might primarily resist scratching through elastic deformation and surface friction. At higher loads, plastic deformation, crack initiation, and even material removal might become more significant. If the batches exhibit different sensitivities to these load-dependent mechanisms, the discrepancy in scratch resistance will increase. However, a future targeted investigation, specifically designed to explore the underlying mechanisms could provide valuable insights for a more precise interpretation of this observed phenomenon.

**Figure 6:**
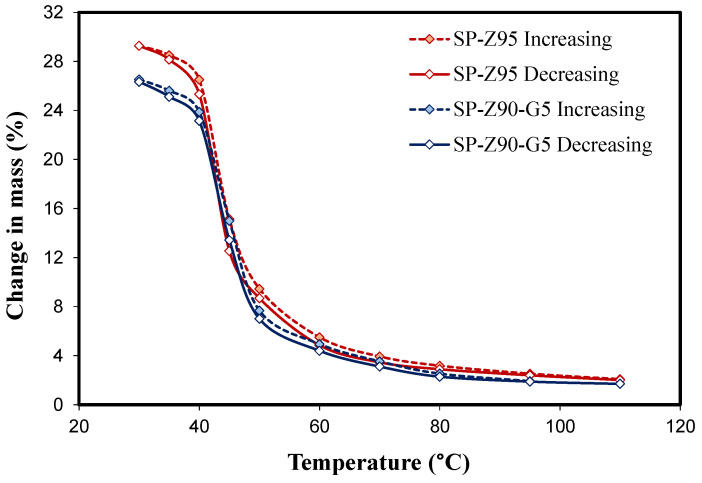
Isobars of adsorption (filled marker) and desorption (empty marker) for composite coatings containing 95 wt.% filler were measured at a water vapor pressure of 11 mbar.

Figure 6 illustrates the water adsorption and desorption isobars for the SP-Z95 and SP-Z90-G5 composite coatings. The data, collected at $p_{H_{2}O}$ of 11 mbar, cover a temperature range from 30 to 110 °C. Filled markers represent adsorption, while empty markers denote desorption. The selected water pressure aligns with Tev approximating 8 °C, typical for optimal cooling performance in adsorption chillers. Batches incorporating 95 wt.% filler served as benchmarks. Analogous results were observed for other compositions, omitted for conciseness.

Consistent with [50], both the composite batches exhibited the distinctive S-shaped adsorption isobar characteristic of SAPO-34 zeolite. Moreover, the adsorption capacities measured for these materials closely aligned with the values documented in the referenced literature [50,51].

Furthermore, the adsorbent composites incorporating a higher amount of zeolite exhibited a significantly enhanced adsorption capacity. This is primarily attributed to the superior adsorption properties of SAPO-34 zeolite, which acts as the primary adsorbent within the composite material. In direct comparison, SP-Z90-G5 composites consistently demonstrated a lower adsorption capacity than SP-Z95 composites. This disparity can be largely explained by the reduced content of the adsorbent material within SP-Z90-G5 composites (90 wt.% compared to 95 wt.% in SP-Z95 batch). Consequently, even at its peak performance, the adsorption capacity of SP-Z90-G5 composites only reached 26.34 wt. %, a level that was comparable to that of commercially available adsorbents commonly employed in sorption technologies [52]. For example, a similar sorption capacity was observed for SAPO-34-based sorbent composites and coatings [23,53,54], confirming its suitability for adsorption heat pump applications. While exhibiting an effective adsorption capacity, SAPO-34’s performance may be surpassed by certain metal–organic frameworks (MOFs), which have demonstrated exceptional adsorption capacities exceeding 50% in some cases, attributed to their high surface area and tunable pore structures, offering promising avenues for further research and optimization in gas adsorption applications [55]. Despite the challenges associated with its high synthesis cost and potential hydrolysis in humid environments, which can affect the long-term performance, more stable zeolite solid sorbents can be considered as preferred option.

In order to better evaluate the reduction in adsorption capacity due to the presence of exfoliated graphite in composite coatings, the maximum water uptake values of the coating, obtained from the isobar curves, were related to the actual amount of zeolite inside the coating. This new parameter is independent of the percentage of zeolite in the coating and was named the Zeolite Maximum Water Uptake (ZMWU). By relating the obtained value of the ZMWU to the maximum water uptake value of pure zeolite (equal to 31.7 wt.% fillers [21]), the efficiency of zeolite within the coating was obtained. These two parameters are reported in Table 2 for coatings with and without graphite.

**Figure 7:**
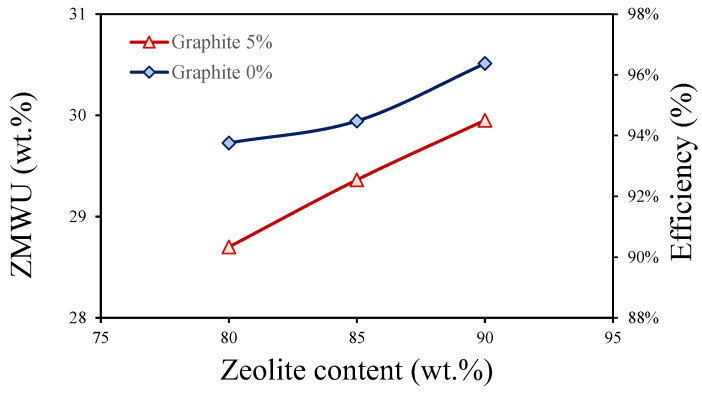
Zeolite Maximum Water Uptake (ZMWU) and efficiency as the zeolite content varies for coatings with and without exfoliated graphite.

It is noticed that both coatings, with and without graphite, had a decreasing value of ZMWU and efficiency as the zeolite content in the coating decreased. This is due to the higher amount of polymer in the coating as the zeolite content decreased, which, despite its permeability to vapor, offered minimal resistance to vapor passage. On the other hand, comparing the values obtained from coatings with and without graphite for the same zeolite content, it can be observed that the presence of graphite caused a slight reduction in efficiency (between 1.9 and 3.4%) and ZMWU (between 0.6 and 1.1 wt.%). This small reduction can be observed more easily in Figure 7, which shows the efficiency and ZMWU values for coatings with and without graphite as the zeolite content changed.

Due to the replacement of 5 wt.% of the vapor-permeable matrix with graphite, the diffusion of water vapor was hindered in the composite coating. This happens because the presence of graphite restricts the participation of zeolite granules in the adsorption process within the coatings, unlike the coatings without graphite, where the same amount of zeolite granules can effectively contribute to the process. Despite this alteration, the reduction in the maximum efficiency remained limited, with a decrease of approximately 3.4%.

Furthermore, it is possible to observe that as the zeolite content increased, the reduction in efficiency caused by the presence of graphite diminished. This improvement can be attributed to the progressive decrease in the sulfonated matrix content. The sulfonated matrix acts as a kinetic barrier, hindering water vapor diffusion. Consequently, the reduction in this barrier with increasing zeolite content suggests that the zeolites themselves become the primary driving force for water vapor diffusion. This implies that the zeolites provide a more efficient pathway for water vapor transport compared to the sulfonated matrix, ultimately enhancing the overall performance. Nevertheless, the performance in the adsorption and desorption processes continues to be excellent even with the inclusion of graphite, highlighting the resilience and reliability of the coating’s ability to interact with water vapor. This finding has significant implications for adsorption processes, as it indicates that optimizing the zeolite content can lead to substantial improvements in performance.

Therefore, the preliminary results suggest that the S-PEEK binder used in this composite adsorbent coating does not provide a relevant adverse water vapor barrier, which could lead to preferential flow pathways and reduced adsorption capacity. However, the addition of the conductive filler does not appear to have a critical negative impact on the adsorption/desorption properties of the zeolite filler. Overall, this class of composite coating, composed of graphite and zeolite fillers embedded in the polymer matrix, shows promise as a potential solution for optimizing the performance of heat exchanger adsorbents and reducing the associated maintenance costs.

The choice to add a graphitic conductive filler can represent an effective approach to improve heat exchange at the interface between the coating and the heat exchanger. A research project is currently in development to assess the potential benefits of incorporating a graphitic conductive filler into a composite coating designed for heat exchangers. The core objective of this laboratory-scale study is to evaluate the influence of the filler on the hydration and dehydration kinetics of the coating, which are relevant factors affecting its thermal properties and performance stability. The experiments are being conducted using a coated heat exchanger of significant dimensions to ensure realistic testing conditions and provide representative data. The results of this study will provide valuable insights into the effectiveness of this approach for enhancing heat exchange at the coating–metal interface.

## 4. Conclusions

The composite coating, SAPO-34/EG/S-PEEK, was investigated to determine its suitability for use in innovative sorption technologies. This was achieved by conducting a series of mechanical and chemo-physical tests. The results of these tests showed that the composite material has good adhesion to the substrate and excellent adsorption properties. In particular,

- The SP-Z90-G5 batch demonstrated suitable adhesive capabilities (average pull-off strength equal to 1.00 MPa), which is consistent with the performances of the other batches. However, its adhesion strength was superior to that of similar composite adsorbent coatings used in AHPs.
- The coatings with exfoliated graphite exhibited lower scratch resistance than those without due to graphite’s lower density and weaker interfacial adhesion with S-PEEK. This effect was more pronounced at higher filler contents (90–95 wt.%). The matrix, which acts as a binding agent, has a key role in maintaining the coating’s structural integrity. At high filler contents, the reduced matrix volume compromises the coating’s cohesive strength, leading to increased scratch sensitivity.
- The SP-Z90-G5 batch, comprising composite coatings with embedded EG, exhibited the highest water absorption capacity, reaching a maximum of 26.34% during the adsorption/desorption experiments. This makes this coating a promising candidate for use in sustainable sorption technologies.

In summary, the SAPO-34/EG/S-PEEK composite coating has shown great potential for use in adsorption heat pumps. It exhibits excellent adhesion to the substrate, high water uptake, and acceptable adhesion properties. These results suggest that this coating could be an effective and efficient material for adsorption applications. To further understand the performance of this material in this application field, further investigation will be devoted to the thermal conductivity measurement and the dynamic adsorption/desorption experiments under varying conditions, including temperature, pressure, and flow rate, in a small-scale coated heat exchanger. This will allow for the evaluation of the kinetic behavior linked to the heat and mass transfer characteristics of the adsorbent material in the adsorber.

## Figures and Tables

**Table 1:** Composite coatings’ composition (wt.%) and coding.

Code	S-PEEK Content (wt. %)	Zeolite Content (wt. %)	Graphite Content (wt. %)
SP	100	0	0
SP-Z80	20	80	0
SP-Z85	15	85	0
SP-Z90	10	90	0
SP-Z95	5	95	0
SP-Z80-G5	15	80	5
SP-Z85-G5	10	85	5
SP-Z90-G5	5	90	5

**Table 2:** Zeolite Maximum Water Uptake (ZMWU) and efficiency for coatings with and without exfoliated graphite.

Code	ZMWU (wt.%)	Efficiency (%)
SP-Z80	29.72	93.76
SP-Z85	29.95	94.48
SP-Z90	30.55	96.37
SP-Z80-G5	28.64	90.33
SP-Z85-G5	29.34	92.54
SP-Z90-G5	29.96	94.5

## Data Availability

The raw data supporting the conclusions of this article will be made available by the authors on request.
